# Abundance and demography of common bottlenose dolphins (*Tursiops truncatus truncatus*) in the Indian River Lagoon, Florida: A robust design capture-recapture analysis

**DOI:** 10.1371/journal.pone.0250657

**Published:** 2021-04-28

**Authors:** Wendy Noke Durden, Eric D. Stolen, Teresa Jablonski, Lydia Moreland, Elisabeth Howells, Anne Sleeman, Matthew Denny, George Biedenbach, Marilyn Mazzoil

**Affiliations:** 1 Hubbs-SeaWorld Research Institute, Melbourne Beach, Melbourne, Florida, United States of America; 2 Department of Biology, University of Central Florida, Orlando, Florida, United States of America; 3 Harbor Branch Oceanographic Institute at Florida Atlantic University, Ft. Pierce, Florida, United States of America; 4 Georgia Aquarium Conservation Field Station, St. Augustine, Florida, United States of America; University of Arkansas Fayetteville, UNITED STATES

## Abstract

Common bottlenose dolphins (*Tursiops truncatus truncatus*) inhabiting the Indian River Lagoon (IRL) estuarine system along the east coast of Florida are impacted by anthropogenic activities and have had multiple unexplained mortality events. Given this, managers need precise estimates of demographic and abundance parameters. Mark-recapture photo-identification boat-based surveys following a Robust Design were used to estimate abundance, adult survival, and temporary emigration for the IRL estuarine system stock of bottlenose dolphins. Models allowed for temporary emigration and included a parameter (time since first capture) to assess evidence for transient individuals. Surveys (*n* = 135) were conducted along predetermined contour and transect lines throughout the entire IRL (2016–2017). The best fitting model allowed survival to differ for residents and transients and to vary by primary period, detection to vary by secondary session, and did not include temporary emigration. Dolphin abundance was estimated from 981 (95% CI: 882–1,090) in winter to 1,078 (95% CI: 968–1,201) in summer with a mean of 1,032 (95% CI: 969–1,098). Model averaged seasonal survival rate for marked residents was 0.85–1.00. Capture probability was 0.20 to 0.42 during secondary sessions and the transient rate was estimated as 0.06 to 0.07. This study is the first Robust Design mark-recapture survey to estimate abundance for IRL dolphins and provides population estimates to improve future survey design, as well as an example of data simulation to validate and optimize sampling design. Transients likely included individuals with home ranges extending north of the IRL requiring further assessment of stock delineation. Results were similar to prior abundance estimates from line-transect aerial surveys suggesting population stability over the last decade. These results will enable managers to evaluate the impact of fisheries-related takes and provide baseline demographic parameters for the IRL dolphin population which contends with anthropogenic impacts and repeated mortality events.

## Introduction

Anthropogenic impacts continue to threaten both coastal and estuarine cetacean populations [1–9], rendering the accurate evaluation of abundance and demographic parameters critical to population management. Various methods have been employed to estimate the abundance of cetacean species, most based on line-transect or mark-recapture methodology [10–15]. Both approaches can provide accurate estimates. While line-transect surveys (aerial and vessel based) are the most utilized methods for coastal species, they only estimate density (abundance) and cannot estimate survival or distinguish between resident and transient animals. In contrast, mark-recapture methods, which rely on patterns of individuals resighted over time, can estimate abundance, survival, and temporary immigration [16,17]. Identifying resident animals within a population is critical, as those animals are most vulnerable to the impacts of repeated anthropogenic activities as well as ecological deterioration in the region.

Common bottlenose dolphins (*Tursiops truncatus truncatus*) inhabiting the Indian River Lagoon (IRL) estuarine system along the east coast of Florida between Ponce Inlet and Jupiter Inlet have been studied for decades and are considered long-term residents comprising the IRL estuarine system dolphin stock [18–20]. The expansive range (~250 km) of this dolphin stock has made vessel-based abundance estimation difficult and prior studies have thus employed line-transect aerial surveys [21–23]. However, more recent studies support the occurrence of transients as well as movements of some individuals that extend beyond the northern boundary of the IRL [21,24–27]. Many cetacean species, including bottlenose dolphins, can be individually identified by naturally occurring markings on the trailing edge of the dorsal fin [28–30]. Photo-identification, or the identification of individuals based on these unique markings, is widely used to study cetaceans [30]. It is commonly combined with mark-recapture methods where marked individuals are “captured” (first identified) and subsequently “recaptured” (resighted) during survey efforts [31–36].

Accurate estimates of abundance and demographic parameters are essential to the management and conservation of the IRL dolphin stock and have become increasingly important as IRL dolphins have experienced multiple Unusual Mortality Events (UMEs) (2001, 2008, 2013; 2013–2015) [37]. During the largest mortality event (2013 UME), a minimum of 77 dolphin mortalities occurred. Based on the mean abundance estimate prior to the event (1,032 dolphins) [21], mortalities represented ~7.5% of the population (19 marked individual mortalities in 2013). Concurrent with this event, the Mid Atlantic UME (2013–2015) coincided and further impacted IRL dolphins [37]. Reoccurring mortality events could indicate serious ecological pressures that may lead to the decline of this stock. Indian River Lagoon dolphins are listed as a strategic stock since anthropogenic mortality likely exceeds Potential Biological Removal set by managers (PBR; the maximum number of mortalities, excluding natural mortalities, that can be removed annually while still allowing the stock to reach or maintain an optimal sustainable population level) [37]. In recent years, the IRL has undergone several large-scale ecosystem changes, most notably phytoplankton blooms that yielded catastrophic seagrass loss [38]. Seagrass meadows have been found to provide critical habitat to prey consumed by estuarine dolphins [39]; therefore, these significant ecological changes are likely to jeopardize the health of the already vulnerable IRL dolphin stock. Recent studies have documented diminished health in IRL dolphins including high concentrations of mercury [40], lingual and genital papillomas [41], and skin disease (lacaziosis) [42,43]. Moreover, interactions with both commercial and recreational fisheries account for up to 12% of the annual mortality [8,44].

The Marine Mammal Protection Act requires dolphin stock assessment, and abundance estimates that are necessary to manage stocks and to calculate the level of sustainable anthropogenic mortality (PBR). Data from aerial surveys and observations of movements of IRL dolphins through inlets have suggested that temporary emigration (movement of resident individuals out of the study area during a portion of the study), and transience (movement of non-resident dolphins through the study area) may have contributed to fluctuating abundance estimates. This is most obvious in portions of the lagoon near inlet access, particularly in response to dramatic declines in water temperature [21,23] which may influence dolphin movement [45–48]. Satellite telemetry has further corroborated oceanic habitat usage in resident IRL dolphins [49]. Photo-identification surveys further support transience occurrence, documenting dolphin movements between the northern portion of the IRL (Mosquito Lagoon) and the St. Johns River (Jacksonville Estuarine system stock-JES), [24,25,50] with a 13% exchange in the individuals examined [25]. Furthermore, genetic research suggests that dolphins inhabiting the Mosquito Lagoon sub-basin may be a disjunct community from the IRL as these animals are genetically distinct from the rest of the IRL and most closely associated with the JES stock [50], suggesting genetic exchange [26,27]. Evaluating transience in IRL dolphins is imperative as parameter estimates will be biased if transients are not considered.

The objectives of this study were to utilize dolphin photo-identification surveys and mark-recapture methodology as components of a Robust Design survey [51] to estimate abundance, survival, occurrence of transients, and temporary emigration of IRL dolphins. Prior to survey initiation, a simulation study was conducted to validate the study design (S1 Text, S1 Fig). We measured transient rates by incorporating a time since first capture parameter into our survival sub-models. Closed population capture-recapture models were used to estimate abundance by sub-basin and primary period (= season).

## Methods

### Ethics statement

Data collection, vessel-based photo-identification surveys of free-ranging bottlenose dolphins, was conducted under permits issued by NOAA Fisheries under General Authorization Letter of Confirmation No.: 16522, 18182, and 20377–01 in tandem with permits issued by Canaveral National Seashore: CANA-2015-SCI-0010 and the U.S. Fish and Wildlife Service: MI-2016-207R. This allowed data collection on the protected/privately owned lands including national wildlife refuges (Cape Canaveral National Seashore, Merritt Island National Wildlife Refuge) and otherwise secured properties (Canaveral Air Force Station, Kennedy Space Center). Data collection did not involve animal handling and thus did not require additional IACUC authorization.

### Study area

**Fig 1:**
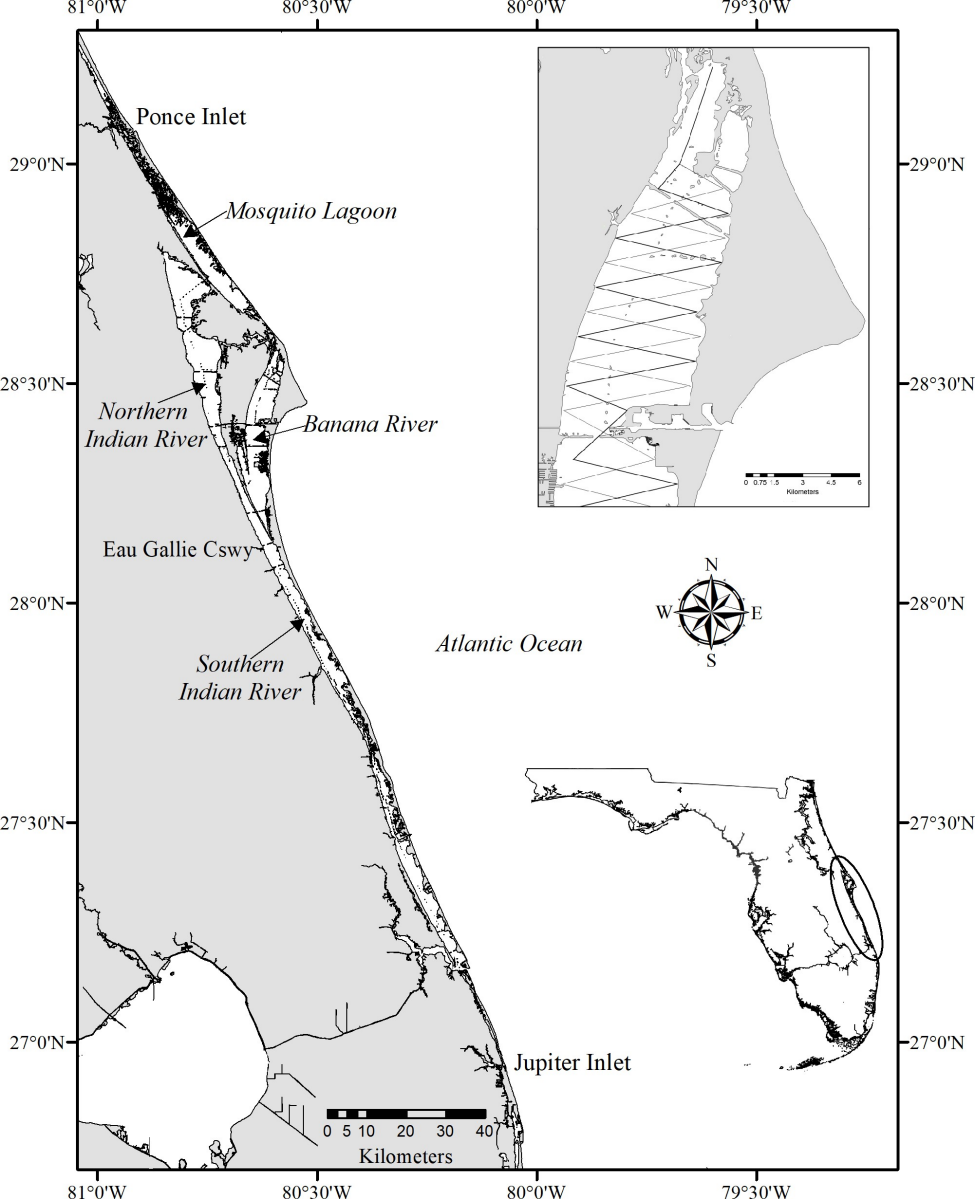
Map of the Indian River Lagoon (Ponce Inlet to Jupiter Inlet; insert lower right) along the east coast of Florida. Sub-basins aligned N-S are: Mosquito Lagoon; Banana River; northern Indian River; southern Indian River. Contour lines and alternating saw-tooth transects were utilized throughout the lagoon as illustrated in the inset portion of the Banana River (upper right). The map was generated using ERSI software, ArcGIS 10.8 (http://www.esri.com/software/argis), using geographic data from Florida Fish and Wildlife Conservation Commission. Reprinted from [Florida Fish and Wildlife Conservation Commission. Florida Marine Research Institute. Atlas of Marine Resources, Version 1.3] under a CC BY license, with permission from [Richard Flamm, Florida Fish and Wildlife Conservation Commission], original copyright [2000].

The IRL is a shallow and diverse estuarine system located along the east coast of central Florida. It opens to the Atlantic Ocean at four inlets and consists of three interconnected basins: the Indian River, Banana River and Mosquito Lagoon [52–54] (Fig 1). The 902 km^2^ estuary spans 220 km with a width of 0.93 to 9.30 km [22] extending from Ponce Inlet to Jupiter Inlet [54]. Although most of the estuary is shallow (<1 m at high tide), depths > 5 m occur in the dredged basins and channels of the Intracoastal Waterway (ICW) [52], which encompasses approximately 2.2% of the lagoon. The IRL in its entirety was divided into four regions (= sub-basins). The Banana River (BR) (202 km^2^) and the Mosquito Lagoon (ML) (140 km^2^) included each sub-basin in its entirety (Fig 1). The Indian River basin was divided into two sub-basins: the northern Indian River (NIR) (378 km^2^, previously defined as north of Eau Gallie Causeway [55], with little tidal and non-tidal flushing [56]), and the southern Indian River (SIR) (182 km^2^) which consisted of three previously defined basins [55] and includes three of the four inlets (Fig 1). Due to a lack of tidal flushing, the BR and NIR have decreased water quality compared to the majority of ML and SIR [56–58], and were the common epicenter of prior IRL UMEs [37,59]. By portioning the lagoon, we were able to compare our results with previous abundance studies [21,23] and with community estimates peripheral to the basins [60].

### Capture-recapture photo-identification surveys

We used a Robust Design photo-identification survey [16,61], which is the preferred method for estimating estuarine dolphin abundance [12,13,51]. The design grouped photo-identification surveys into four short-duration (less than three weeks) primary periods, each containing three replicate surveys (secondary sessions), during which population closure was assumed. The four primary periods were separated by longer duration time intervals (~three months) during which an open population was assumed. The Robust Design estimates abundance using closed mark-recapture models based on recaptures of individuals between the secondary sessions, and survival and temporary immigration/emigration using open mark-recapture models based on recaptures of individuals between the primary periods. Vessel based capture-recapture surveys were conducted in the IRL between August 2016 and May 2017, with the following assumptions: 1) Population closure within a primary period and emigration/immigration between primary sampling periods being temporary; 2) Dorsal fin marks were unique, permanent, and correctly identified; 3) Individual capture probabilities were equal within a secondary session, and 4) Individual survival probabilities were equal among individuals within a primary period [16,61,62]. To account for the potential occurrence of non-resident individuals, we included a transient parameter by modeling survival for individuals during the interval following the primary period of their first sighting differently than for subsequent intervals. Additional assumptions were that marked and unmarked animals did not differ in detection, movement, or survival parameters, and mixed randomly [16,61,62]. We defined four primary periods (summer = June-August, fall = September-November, winter = December-February, and spring = March-May) [63]. Primary periods contained three secondary sessions that were completed under optimal conditions with a Beaufort Sea State ≤3 (conditions = glassy to crests of some large wavelets breaking), in the shortest time period to meet the assumption of closure (target: ≤3 weeks). Secondary sessions (complete survey of the IRL) were separated by at least one day to allow for population mixing [13]. Existing dorsal fin catalogs were utilized and established protocols were closely followed [51]. The survey design used both depth contour lines and alternating saw-tooth transects (total length ~ 743 km) to minimize capture heterogeneity (Fig 1). Alternating saw tooth transects (2.5 km apart) were traversed in areas where lagoon width exceeded 1.25 km in either direction from the N-S contour line (based on 95% of prior sightings) [64]. Predetermined routes were downloaded into a Global Positioning System unit to ensure transect adherence. Each secondary survey covering the IRL bottlenose dolphin stock (Ponce Inlet-Jupiter Inlet) [65] was completed within one to three days (Fig 1).

Vessels (5–7 m) containing researchers (*n* ≥3) traveled at 10–12 knots to search along pre-determined tracks. Dorsal fins of encountered dolphins were photographed using a Canon EOS digital camera with a 100–400 mm telephoto lens (Canon USA, Inc., Melville, NY, USA). A dolphin group (= sighting) was defined as all dolphins within 100 m that were engaged in similar behavior with the same general heading [66]. Calves were identified as <75% adult size and swimming in adult echelon (i.e., in proximity to adult mid-lateral flank). Young-of-year (YOY) were <50% adult size, swimming in echelon, and characterized by: (a) dark coloration; (b) floppy dorsal fin; (c) presence of fetal lines; (d) extreme buoyancy; and (e) rostrum-first surfacing [67–69]. Animals not identified as calves or young of the year were considered adults. Additional data included: (a) time-location-GPS readings; (b) lat-long in decimal degrees; (c) behavior; (d) estimated water depth; (e) estimated group size/composition; (f) environmental covariates; and (g) sighting conditions (Beaufort Sea state, chop height, glare).

### Photo-identification analyses and cumulative curve

Dorsal fin image analyses followed established protocols [70]. Briefly, marked dorsal fins were sorted by notch patterns, with the best photograph serving as the ‘type’ for each dolphin. Subsequently, unambiguous matches were accepted as re-identifications if a minimum of two experienced personnel agreed. A new individual was added to the catalog if distinctly marked but not matched with an existing photo.

To minimize false matches, images were graded for photographic quality based on a weighted scale of five characteristics [71]. Images were then assigned a quality score as follows: Q1 = excellent; Q2 = average; Q3 = poor. Dorsal fin distinctiveness was assigned a rating as follows: D1 = very distinct, D2 = moderately distinct, at least two features or one major feature; and D3 = not distinct, few to no features [71,72]. To avoid potential identification errors, only Q1 and Q2 quality photographs were used as individual detections in capture-recapture analyses. Thus mark-recapture estimates only applied to the identifiable individuals (D1 and D2).Unidentifiable individuals (D3; not meeting D1, D2 criteria), were used to estimate the proportion of marked individuals and, as discussed below, the total population size of an area. Because of the potential for changes in distinctiveness, non-independence from mothers, and other causes of capture heterogeneity, calves and YOY were excluded from capture-recapture analyses [51]. Dolphin survival can vary greatly by age class [73]; therefore, focusing on only adults ensured less capture heterogeneity and more reliable survival estimates.

To informally evaluate population closure, a cumulative curve of marked individuals (i.e., discovery curve) [74] was plotted based on the cumulative total number of distinct (D1 andD2) dolphins across each secondary session. Lastly, the percent of exchange of marked dolphins between sub-basins was evaluated.

### Robust design models

We used program MARK [75] via package RMark [76] in R [77] to assess various Robust Design capture-recapture models. For each primary period, parameters estimated included dolphin abundance (*N*), the probability of apparent survival (φ), the probability of detection (p), and two temporary emigration parameters: the conditional probability of an animal not being available for capture (e.g., outside of the study area or within the study area but not available for detection) given that it was available (ϒ’’) or not available (ϒ’) during the previous primary period. Some models also included a time since initial capture (“transient”) parameter to allow separate estimates of survival for animals captured in more than one primary period-season (= residents) [78].

A total of 36 models were fit with combinations of structural covariates for detection, survival, and temporary emigration parameters. Detection models allowed detection to vary between primary periods (p(season)), secondary sessions (p(season*session)), or to be equal across all sampling occasions (p(.)). The probability of first detection and the probability of recapture was assumed equal for all models (no behavioral response). Survival was modeled as varying by primary period (ϕ(season)) or as a constant (ϕ(.)). A transient covariate was also included in some models to allow survival during interval following the primary period of an individual’s initial capture to be estimated separately from survival for all subsequent primary periods [78]. For those models, survival was allowed to vary by primary period (ϕ(transient*season)) or to be constant (ϕ(transient)). We assumed that transients were likely to be sighted in only one primary period and the set of dolphins captured for the first time in any primary period was a mixture of transients and residents. Therefore, survival estimates for the first primary period after initial capture (φ_1_) included apparent mortality (due to permanent emigration) and were likely biased low [79]. The survival estimates for the remaining observations (φ_2_) more accurately reflected resident survival since animals seen in more than one primary period (season) were likely residents. Assuming that the transients and residents had the same detection probability within the relatively short primary periods (and thus that the transients left between the primary periods), then the proportion of transients among the newly-marked animals (distinct dolphins seen for the first time) can be calculated as τ_i_ = φ _i2_/φ _i1_ where the i subscript refers to the primary period at the beginning of the survival interval. Although the number of transients could be computed as T_i_ = N_i_/(N_i_ + m_i_), where N_i_ = the number of newly-marked individuals and m_i_ = the number of previously marked individuals captured within time period i [79,80], we did not adjust abundance estimates by removing estimated transients. Temporary emigration was modeled as: 1) Markovian movement in which the probability of availability was dependent on the previous state (available or unavailable) (ϒ’’(.) ϒ’(.)); 2) random movement in which availability probability did not differ based on the previous state (ϒ’’ = ϒ’(.)); or 3) no movement models (ϒ” = ϒ’ = 0) with no temporary emigration. For Markovian and random movement models, the temporary emigration parameters were constrained to be constant throughout the study. Attempts made to model temporary emigration as varying over time resulted in some parameters not being identifiable when the transient covariate was included, potentially due to the number of primary periods (*n* = 4) [81]. We chose to constrain movement parameters to be equal over time for all models with temporary emigration as a conservative approach to comparing models.

The robust design lacks an overall goodness-of-fit test, therefore to evaluate model fit we used Fletcher’s generic goodness-of-fit statistic (c-hat) [82,83] calculated by program MARK for the two most parameterized models. This included both time (season) and “transient” (time since initial capture) effects on survival, time-specific (season and session) detection parameters, and random temporary emigration parameters. The assumption of population closure during primary periods was evaluated using goodness-of-fit statistics implemented in program Close Test version 3 [84]. Model comparisons were based on the small-sample adjusted Akaike Information Criterion AICc [85], calculated with the theoretical number of estimable model parameters (i.e., the column rank of the design matrix), rather than the estimated rank of the variance–covariance matrix. Models with ΔAICc < 10 were evaluated for evidence of numerical estimation errors (indicating a lack of parameter identifiability). Akaike weights were reported and convey the relative support compared to all candidate models for each model on a scale of zero to one [85]. Akaike weights and final abundance estimation were based on model sets including only similar movement sub-model types [85], excluding models with numerical estimation problems for some parameters (S1 Table).

To obtain abundance estimates for the total population (identifiable, D1+D2 and unidentifiable, D3 dolphins) an *ad hoc* adjustment was applied as follows [78]. The proportion of identified individuals (Ɵ) was calculated as the number of adult individuals that were marked (D1 +D2) divided by the total number of adult individuals (D1 + D2 + D3) observed during each primary period (season). Abundance estimates for the total population $\left({\hat{N}}_{total}\right)$ in each primary period (season) were then calculated by dividing the population estimates for identified individuals $\left(\hat{N}\right)$ by the proportion marked (Ɵ). The variance of each total population estimate was calculated as:

$$var({\hat{N}}_{total})={\hat{N}}_{total}^{2}\cdot (\frac{var(\hat{N})}{{\hat{N}}^{2}}+\frac{1-\hat{Ɵ}}{n\cdot \hat{Ɵ}})$$

where *n* was the number of dolphins from which Ɵ was estimated. Log-normal 95% confidence intervals for total population size was calculated with a lower limit of ${\hat{N}}_{total}$/C and an upper limit of ${\hat{N}}_{total}$·C, where

$$C=exp\;[z_{\frac{\alpha }{2}}\cdot \sqrt{ln(1+{\left[CV({\hat{N}}_{total})\right]}^{2}})]$$

in which z is the normal deviate, CV is the coefficient of variation, and α = 0.05. Density was estimated as dolphins/km^2^ by dividing total abundance as calculated above by IRL area: (902 km^2^).

### Closed sub-basin abundance models

To obtain seasonal abundance estimates ($\hat{N}$) for each of the four sub-basins, closed population capture-recapture abundance models were fit with program MARK [75] via package RMARK [76] in R [77]. These capture-recapture abundance models made the following assumptions: population closure (no births, deaths, immigration or emigration), unique marks that were permanent and correctly read, and equal capture probability for marked and unmarked animals (random mixing after first capture) [86,87]. A separate model was fit to each sub-basin by primary period (season), which allowed each secondary session to have a unique capture probability [88]. Multi-state robust design capture-recapture models could potentially have provided sub-basin-specific abundance estimates (and movement probabilities), but these models require many additional parameters. Given that data were limited to four primary periods [81], such complex models were prohibited (too many parameters for the available data). Although potentially losing some statistical power, fitting separate closed models produced reasonable abundance estimates with good precision and provided valuable baseline estimates for resource managers. Closed population capture-recapture abundance estimates were adjusted for unidentifiable individuals following the methods described above for Robust Design abundance estimates. Density by sub-basin was estimated as dolphins/km^2^ by dividing the total abundance estimates by the area of each sub-basin.

## Results

### Field effort and photo-identification

From August 2016 through May 2017, 135 vessel surveys (25 survey days) were conducted throughout the IRL during four capture-recapture primary periods (12 secondary sessions). Each secondary session (complete IRL survey replicate) employed 11–13 vessels (11.42 ± 1.00 SD) over 1–3 d (2.3 ± 0.65 SD). Each primary period was completed in 13–36 d (20.0 ± 10.9 SD). Vessel surveys ranged from 3.28–14.25 h (8.72 ± 2.21 SD; total field hours: 1,177.58 h). Over 159,000 photographs were taken of 1,465 dolphin groups, totaling 5,973 dolphins (S2 Table). Dorsal fin images used in analyses were Q1 = 87.3% or Q2 = 8.4%, while Q3 = 4.3% were excluded. Calves and YOYs comprised 19.2% of animals sighted. Mean group size was 4.08 (± 4.24 SD) (Table 1).

**Table 1:** Mean group size (± SD) of bottlenose dolphins in the Indian River Lagoon and its four sub-basins by primary period.

Period	Indian River Lagoon	Mosquito Lagoon	Banana River	Northern Indian River	Southern Indian River
Summer	5.08 ± 5.10	4.27 ± 4.85	6.58 ± 6.05	5.39 ± 5.13	4.40 ± 3.70
Fall	3.68 ± 3.98	3.49 ± 3.43	3.46 ± 3.04	3.83 ± 4.28	4.01 ± 5.04
Winter	3.69 ± 3.73	2.95 ± 2.89	5.26 ± 5.61	3.21 ± 3.21	3.55 ± 2.58
Spring	3.94 ± 3.98	3.01 ± 3.28	4.52 ± 5.00	4.33 ± 4.19	3.83 ± 2.74
Mean	4.08 ± 4.24	3.49 ± 3.81	4.92 ± 5.15	4.10 ± 4.21	3.88 ± 3.56

### Cumulative curve of marked individuals and distribution patterns

**Fig 2:**
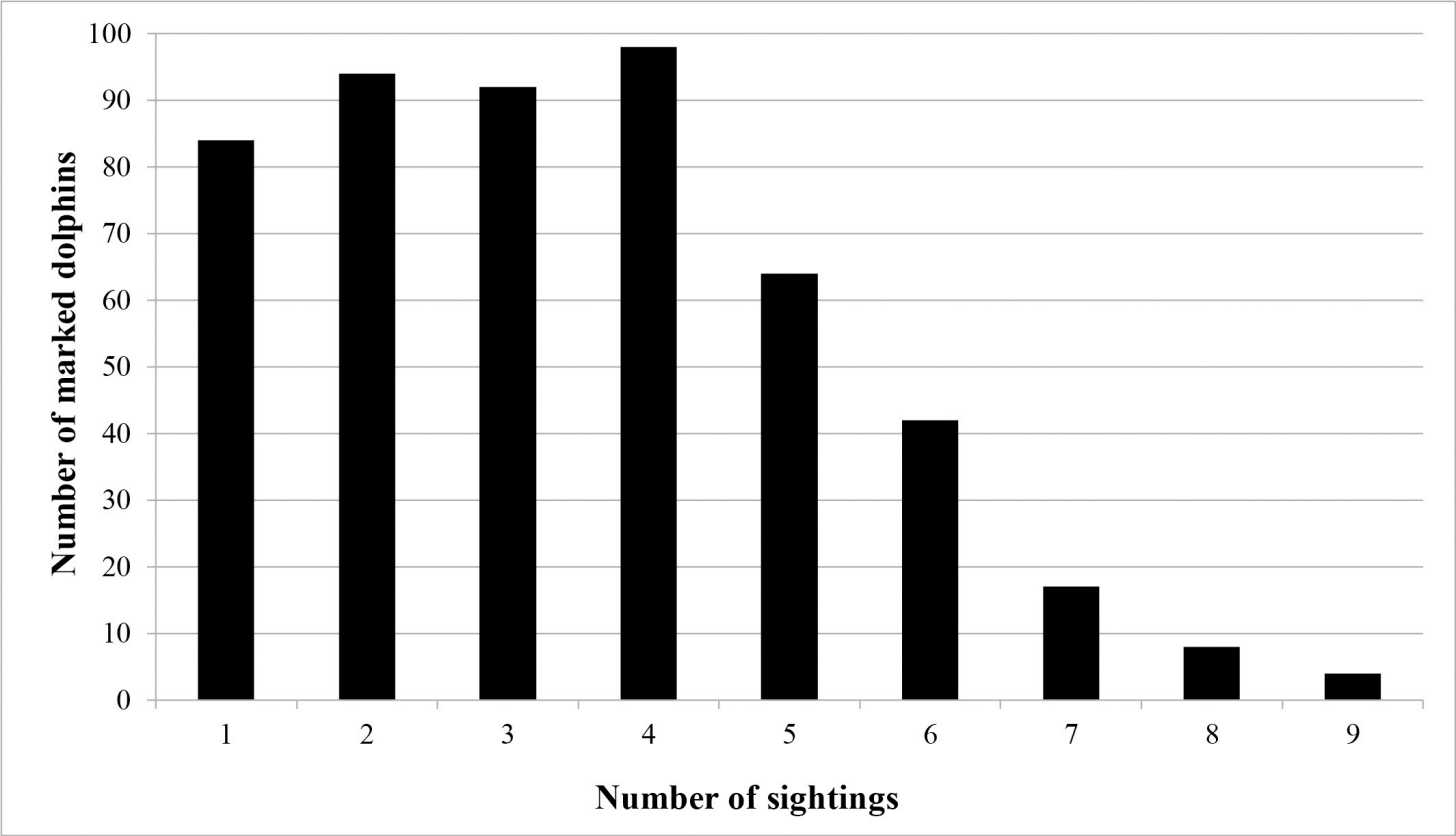
Sighting frequency of marked bottlenose dolphins in the Indian River Lagoon (Florida east central coast). Number of sightings (= number of secondary sessions out of 12) in which an individual was photographed.

**Fig 3:**
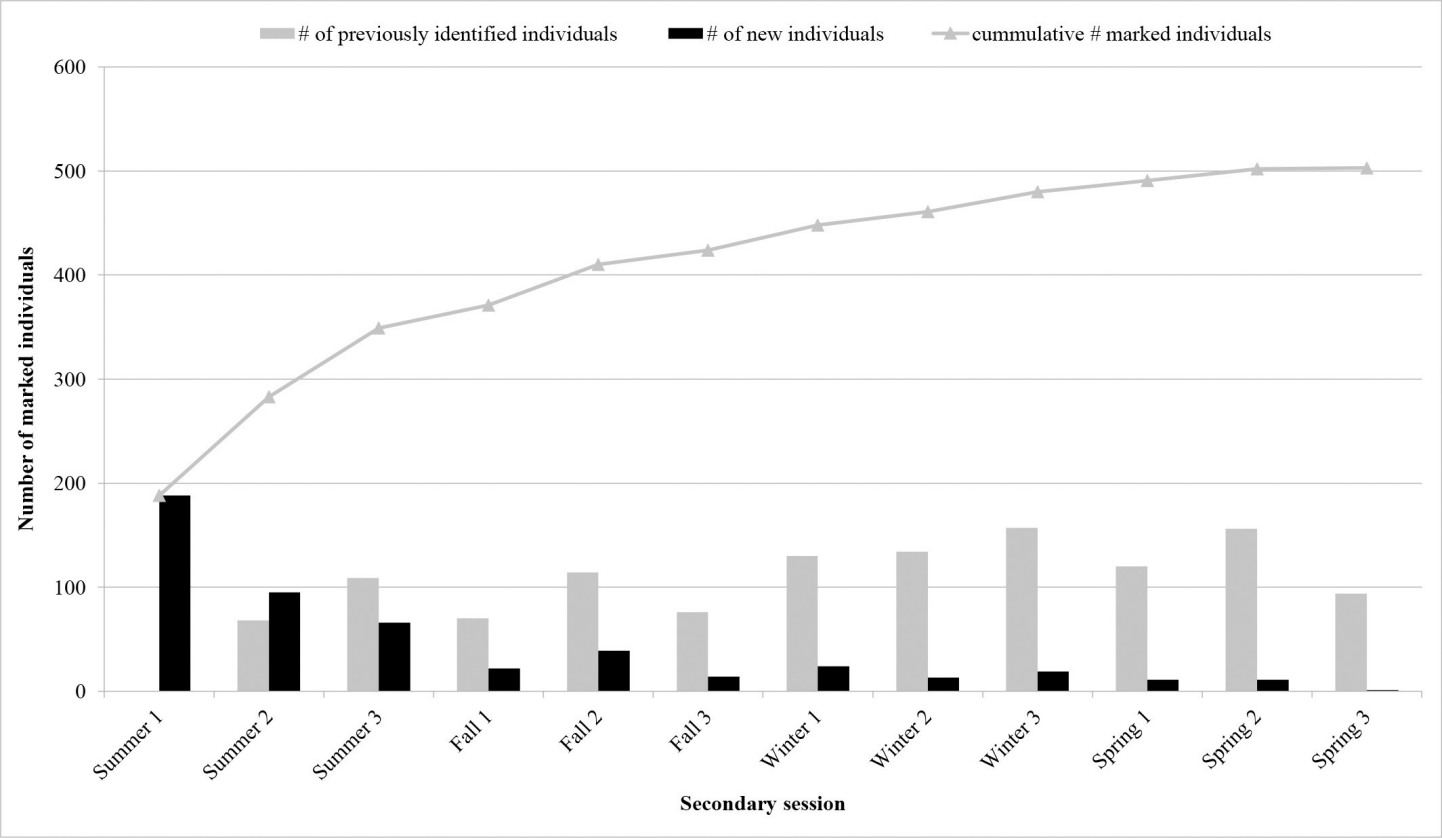
Number of marked bottlenose dolphins by seasonal replicate (= secondary session) during Indian River Lagoon surveys (Florida east-central coast). Superimposed onto the plot is the cumulative curve of distinct (D1) and moderately distinct (D2) individuals.

Distinctively marked individuals (*n* = 503) were recorded, with 369 (73.4%) observed in one sub-basin. The southern Indian River had the greatest number with 124 (33.6%) followed by 115 (31.2%) in Mosquito Lagoon, 73 (19.8%) in the northern Indian River and 57 (15.4%) in the Banana River. A total of 112 marked individuals (22%) were seen in two sub-basins. Movements between three sub-basins were only observed between the NIR, BR and SIR, 22 individuals (4%). Of the individuals seen in two sub-basins, the greatest exchange occurred between the northern Indian River and the Banana River, followed by the northern Indian River and the southern Indian River (Table 2). Eighty-seven percent of the distinct individuals in Mosquito Lagoon were only observed there, while the remaining 13% were also observed in the northern Indian River (Table 2). Of the 503 distinctly marked animals, 84 (16.7%) were sighted during a single survey only (Fig 2). These 84 animals were distributed across all primary periods (fall = 17; spring = 18, summer = 29, and winter = 20) and sub-basins (Mosquito Lagoon = 23, Banana River = 17, northern Indian River = 22, southern Indian River = 22). Frequency of sightings of marked animals over the four seasons was one (*n* = 105; 20.9%), two (*n* = 155, 30.8%), three (*n* = 168, 33.4%) and four seasons (*n* = 75, 14.9%), and over the 12 secondary sessions frequency ranged from one to nine (Fig 2). Over the 12 secondary sessions, cumulative captures of marked animals increased steadily and plateaued over the final sessions (Fig 3). Within primary periods, a sharp decline in the number of new marked animals between sessions two and three was evident for nearly all periods (Fig 3).

**Table 2:** Percentage of marked bottlenose dolphins exchanged among four Indian River Lagoon sub-basins.

Exchange sub-basin	Sub-basin of origin			
	Mosquito Lagoon	Banana River	Northern Indian River	Southern Indian River
Mosquito Lagoon	87	0	8	0
Banana River	0	36	28	18
Northern Indian River	13	38	35	27
Southern Indian River	0	26	29	55

Each row gives the percent of all the individuals that were initially photographed in one sub-basin (origin) that were also photographed in another sub-basin (exchange).

### Robust design and model selection

Fletcher c-hat statistic was 1.03 for the two most parameterized models, indicating no significant overdispersion in our data, thus models were not adjusted for overdispersion. The best supported models (12 of the 36) had AICc values <4 (S1 and S3 Tables). Of these, five had ΔAICc < 2 (= high support). The remaining seven had 2 < ΔAICc < 4 (= moderate support) [89]. Population closure during primary periods was supported except during spring when potential losses between secondary sessions one and two were indicated (S4 Table). The best supported model allowed survival to vary by primary period (season) and by time since initial capture (“transient”), and no temporary emigration (S3 Table). The second-best model also allowed survival to vary by time since initial capture but had random temporary emigration. All of the best supported models allowed a unique detection probability estimate for each secondary session within seasons. Model average estimates of detectability ranged from 0.20 to 0.42 between secondary sessions (Table 3, S2 Fig). The model average estimates of seasonal (three month) marked dolphin survival for residents ranged from 0.85–1.00 (Table 4).

**Table 3:** Model averaged estimates of detection (p) of marked bottlenose dolphins during Indian River Lagoon surveys (Florida east-central coast) by seasonal replicate (= secondary session).

Session	p	SE	95% CI	
			LCL	UCL
Summer session 1	0.42	0.03	0.36	0.47
Summer session 2	0.36	0.03	0.31	0.41
Summer session 3	0.39	0.03	0.34	0.44
Fall session 1	0.21	0.02	0.17	0.26
Fall session 2	0.35	0.03	0.29	0.41
Fall session 3	0.20	0.02	0.17	0.25
Winter session 1	0.34	0.03	0.29	0.40
Winter session 2	0.33	0.03	0.28	0.38
Winter session 3	0.39	0.03	0.34	0.45
Spring session 1	0.32	0.03	0.26	0.38
Spring session 2	0.40	0.04	0.34	0.48
Spring session 3	0.23	0.03	0.18	0.28

Detection was estimated with Robust Design capture-recapture models for Indian River Lagoon dolphins (2016–2017). *LCL = Lower confidence limit; UCL = Upper confidence limit.

**Table 4:** Model averaged estimates of adult survival rates (S) of marked bottlenose dolphins between primary periods during Indian River Lagoon surveys (Florida east-central coast).

Survival	Random S (95% CI)	SE	No movement S (95% CI)	SE	Markovian S (95% CI)	SE
S transient Summer-Fall	0.94 (0.88, 0.97)	0.02	0.93 (0.88, 0.96)	0.02	0.95 (0.83, 0.99)	0.03
S resident Fall-Winter	0.98 (0.74, 1.00)	0.03	0.98 (0.74, 1.00)	0.03	1.00 (1.00, 1.00)	0.00
S transient Fall-Winter	0.93 (0.74, 0.98)	0.05	0.90 (0.69, 0.98)	0.06	0.94 (0.67, 0.99)	0.06
S resident Winter-Spring	0.92 (0.60, 0.99)	0.08	0.85 (0.64, 0.94)	0.07	0.93 (0.53, 0.99)	0.08
S transient Winter-Spring	0.90 (0.61, 0.98)	0.08	0.84 (0.57, 0.95)	0.09	0.90 (0.59, 0.98)	0.08

Model-averaged survival estimates are given separately for each of the three temporary emigration models (random, no movement, and Markovian-see methods). Transient refers to survival estimates for the first captures (including transients and residents) while resident refers to survival estimates for all subsequent captures (residents only). SE = standard error.

Temporary emigration models with random movement (combined Akaike model weight = 0.42) had more support than those with no movement (combined Akaike model weight = 0.34) or Markovian movement (combined Akaike model weight = 0.22) (S1 Table). Based on model average estimates of Markovian models, the probability that a dolphin became unavailable if it had been available in the previous primary period (ϒ”) was quite low with the model average estimate being 0.05 (SE = 0.05). The probability was greater that a dolphin remained unavailable for observation (outside the study area) given that it had been unavailable in the previous primary period (ϒ’), but estimates lacked precision 0.48 (SE = 0.44). The estimated proportion of transients (transient rate) among the marked population ranged from 0.06 in winter to 0.07 in fall, the only seasons for which the proportion of transients could be estimated. The proportion of marked individuals ranged from 0.40–0.45 between primary periods (Table 5). Abundance for the IRL Estuarine System stock ranged from 981 (95% CI: 882–1,090) dolphins in the winter season to 1,078 (95% CI: 968–1,201) dolphins in the summer season (Table 5, S3 Fig). Mean estimated dolphin abundance was 1,032 (95% CI: 969–1,098) (Table 5). IRL density ranged from 1.09–1.20 dolphins/km^2^ (1.15 ± 0.05 SD) (Table 5).

**Table 5:** Estimated abundance (95% CI) by primary period for the Indian River Lagoon dolphin population (2016–2017).

Primary period	$\hat{N}$ identified (95% CI)	Proportion identified	$\hat{N}$ identified + unidentified (95% CI)	Density (dolphins/km^2^)
Summer	450 (421, 491)	0.42	1078 (968, 1201)	1.20
Fall	447 (400, 512)	0.43	1040 (903, 1198)	1.15
Winter	445 (411, 492)	0.45	981 (882, 1090)	1.09
Spring	408 (367, 468)	0.40	1027 (895, 1178)	1.14

Abundance of marked fraction ($\hat{N}$ identified) was the model averaged abundance estimates for dolphins with identifiable dorsal fins. $\hat{N}$ identified + unidentified was the estimated number for all adult dolphins after adjusting for the proportion identified.

### Abundance by sub-basin (closed models)

The greatest mean abundance was in the southern Indian River, followed by the Banana River and northern Indian River; while the lowest mean abundance was observed in Mosquito Lagoon (Table 6, S4 Fig). Mean estimates of detectability were greatest for the Banana River (0.40) and Mosquito Lagoon (0.32), followed by the Northern Indian River (0.23) and the Southern Indian River (0.20) (S5 Table). Dolphin density (dolphins/km^2^) varied by sub-basin with the largest mean density in the southern Indian River (2.00 ± 0.60 SD), followed by the Banana River (1.72 ± 0.57 SD), Mosquito Lagoon (1.23 ± 0.32 SD), and the northern Indian River (0.91 ± 0.27 SD) (Table 6).

**Table 6:** Estimated abundance (95% CI) and density of bottlenose dolphins in the Indian River Lagoon sub-basins, by primary period during 2016–2017.

Sub-basin	Primary period	N all	N identified	Proportion identified	$\hat{N}$ identified (95% CI)	$\hat{N}$ identified and unidentified (95% CI)	Density (dolphins/km^2^)
ML	Summer	445	229	0.51	105 (91,133)	205 (166, 252)	1.46
ML	Fall	255	105	0.41	54 (41, 86)	130 (87, 194)	0.93
ML	Winter	281	127	0.45	69 (61, 89)	153 (122, 191)	1.09
ML	Spring	218	109	0.50	113 (87, 168)	225 (157, 324)	1.61
BR	Summer	427	140	0.33	112 (104, 127)	341 (288, 403)	1.69
BR	Fall	232	69	0.30	143 (107, 216)	482 (321, 725)	2.39
BR	Winter	410	96	0.23	86 (77, 107)	369 (290, 469)	1.82
BR	Spring	333	106	0.32	65 (61, 77)	204 (168, 248)	1.01
NIR	Summer	356	140	0.39	193 (148, 275)	491 (349, 690)	1.30
NIR	Fall	173	62	0.36	109 (74, 192)	303 (178, 517)	0.80
NIR	Winter	298	107	0.36	94 (78, 127)	260 (195, 348)	0.69
NIR	Spring	287	117	0.41	134 (107, 188)	329 (239, 452)	0.87
SIR	Summer	232	153	0.66	147 (115, 208)	223 (163, 305)	1.23
SIR	Fall	262	149	0.57	270 (195, 411)	475 (320, 705)	2.61
SIR	Winter	364	207	0.57	240 (201, 303)	421 (336, 528)	2.32
SIR	Spring	227	126	0.56	188 (132, 304)	338 (216, 527)	1.86

Abundance estimates ($\hat{N}$) calculated using closed capture-recapture models are given for the dolphins with identifiable dorsal fins (identified), and the estimated total for all adult dolphins after adjusting for the proportion of unidentified animals observed in each primary period (identified and unidentified). The raw number of identified adults (N identified) and all adults (N all) observed during each season is also presented. Sub-basins included the Mosquito Lagoon (ML), Banana River (BR), northern Indian River (NIR) and southern Indian River (SIR). 95% confidence intervals of the estimates are given in parentheses.

The mean percent of identified individuals 43 ± 0.12 SD varied between sub-basins with the largest occurring in the southern Indian River (58.8%), followed by Mosquito Lagoon (47.0%), the Northern Indian River (38.0%) and the Banana River (29.4%) (Table 6).

## Discussion

Estimating the abundance and demography of bay, sound and estuarine bottlenose dolphin populations pose challenges due to shallow water inaccessibility, the uncertainty of population boundaries, and the movement of non-residents within the system [1–3,13,90,91]. Here we implemented a Robust Design photo-identification survey to estimate bottlenose dolphin abundance for the Indian River Lagoon estuarine system stock (Florida) while accounting for both temporary emigration by residents and occurrence of transient (non-resident) dolphins. Our estimates achieved good precision and were comparable to those derived from recent IRL studies [21], suggesting that the population size has been stable over the last decade. Additionally, our study provided estimates of high seasonal survival, low transient rate and moderate temporary emigration rate. Model selection preference for models without temporary emigration (the ’no movement’ model) and the low estimated transient rate supported a resident IRL dolphin stock, in agreement with results from prior studies [18,19]. While the observed transient rate was low, by excluding potential transients we were able to more accurately estimate resident IRL dolphin survival by separating dolphins sighted in only one season (= transients) from those sighted in more than one season. These data provide critical guidance for a population whose diminishing health, coupled with anthropogenic impacts, likely promotes a mortality rate that exceeds potential biological removal [37].

### Demographic and abundance parameters

Our capture-recapture study estimated a transient rate of 7%, which was less than the observed rate of single sightings (e.g., 84 of 503 marked dolphins were sighted only once, with 27% of these in Mosquito Lagoon). This illustrates the utility of the model allowing for transients to be separated from residents that were potentially only seen once. The low estimate of transient rate further supported the use of a closed Robust Design. Within primary periods, a sharp decline in the number of new marked animals between sessions two and three was evident for nearly all periods (exception was a slight winter increase; 13 to 19 new marked animals between sessions), supporting population closure within the primary periods [74]. Furthermore, the majority of those that were sighted only once in Mosquito Lagoon (57%) also inhabited the adjacent Halifax River estuary to the north, and a few ranged further north into the JES (S6 Table) [25]. However, while this low rate suggests transients did not play a large role in the population biology of IRL dolphins, results should be interpreted with the understanding that rates depend on how study boundaries were defined which likely did not perfectly align with biological factors. Furthermore, transient rates may be driven by environmental conditions, as found in a recent study [24]. Although rare (4.3%), it is possible that excluded poor quality images contributed to some resident dolphins being categorized as transients. Since apparent survival is a product of true survival and site fidelity [92], it was expected that the inclusion of a transient parameter would increase survival rates for marked IRL adults by reducing the effect of non-residents on apparent survival (i.e., reducing negative bias). However, including the transient parameter may have over corrected and biased resident survival upward by removing individuals from those estimates with the lowest survival [93]. This could be reduced in future studies by including covariates that identify dolphins known to range beyond the study area.

Evidence was mixed for the occurrence of temporary emigration, but model selection generally supported non-structured movement (i.e., random), suggesting that some dolphins were, at times, unavailable for re-sighting. Although less supported, the Markovian movement model indicated that while IRL dolphins rarely temporarily emigrate from the study area, those that do may remain absent for more than one season. Likewise, we found the probability of an individual not being available during a primary period if it was not available in the prior session was greater (48%) than if it was available in the prior session (5%). Temporary emigration, as measured by the robust design, can be caused by true absence from the study area, or by individuals present but unobservable during a primary period. Individuals inhabiting the periphery of the study region, with home ranges extending beyond the IRL border, may have contributed to such temporary emigration. Resident dolphins have also been documented utilizing oceanic habitat [49]. Availability bias [94] may also occur due to dolphins utilizing the labyrinth of canals, freshwater creeks, and shallow waters surrounding islands throughout the IRL [49,64]. This bias could be potentially mitigated by including covariates that model detection heterogeneity such as distance from shallow labyrinth or inlets.

Survival rates for identified residents varied widely across movement models. A trend of initially high resident survival, which decreased between the first (fall-winter) and the second (winter-spring) interval, was evident across all models that included both transient and season effects. While variation in survival between seasons may have occurred, the reduction may instead be due to the limited number of primary periods employed. Low-biased survival at the end of a study (the last survival estimates in a time series) has been noted in prior studies, and is a potential result of constraints or biased estimates at the end of the time series [81]. During our study, cases of documented marked adult mortality were low (*n* = 3, S7 Table), although may be underrepresented since even in the well-studied Sarasota Bay estuary, only one-third of dolphin carcasses were recovered [95]. Marked resident survival in the first (fall-winter) time period, range 0.98–1.00 more closely matched observed mortality. Thus, the first time period likely provides the most reliable estimate of survival for utilization by resource managers. Longer-term studies with increased primary periods should improve survival estimation.

Detection probability between secondary sessions ranged from 0.20–0.42, values which fell within recommended bounds of effective capture-recapture survey designs (0.2–0.3; [51]). Detection was least in fall, and likely influenced by an increased sea state (waves) common to that season [96]. The mean proportion of marked dolphins (0.43 ± 0.12) was less than other study systems with mean rates of 0.79 and 0.72 respectively [13,97]. Differences in dorsal fin marking rates between populations may be influenced by ecosystems [98]. Since extensive portions of the IRL are relatively isolated [56] in contrast to more open bays [2,13,90,91], heterogeneity between regions (e.g., anthropogenic activities, conspecific interactions) could contribute to differences in marked ratios. Furthermore, in other study regions, rates of dorsal fin marking have a sex-bias, with significantly higher rates of dorsal fin nicks in males [99]. Potential heterogeneity in capture probability may occur as larger groups contain more calves [100], and by extension female dolphins with less propensity to be marked [99]. Interestingly, the marked ratio varied by sub-basin, with the greatest in the southern Indian River (0.588) and the least in the Banana River (0.294). Dolphins in the southern lagoon have the highest prevalence of boat-injuries that yield dorsal fin disfigurement, in relation to other sub-basins [101], and this may contribute to variability in marks among sub-basins. Much of the northern Banana River prohibits motorized vessels and has no public usage [102], thereby limiting entanglement, vessel strikes, and capture-release activities for dolphin health assessment that may influence dorsal fin marking [8,101,103].

### Abundance and distribution patterns

Our mean dolphin abundance and density estimates for the Indian River Lagoon stock were similar to those derived from multi-year line transect aerial surveys [21]. Both the current study and prior aerial efforts [21] observed the greatest mean abundance in the southern Indian River sub-basin and the lowest in Mosquito Lagoon. Only slight changes in abundance were observed between seasons, in contrast to the aerial surveys, in which dolphin abundance was greater in winter than summer [21]. Winter aerial surveys were conducted during several unusually cold periods, which may have contributed to increased abundance in the Mosquito Lagoon and the southern Indian River [21]. While aerial survey estimates of dolphin detectability did not vary seasonally [21], these extreme cold events possibly influenced abundance estimates through heterogeneity in availability due to dolphin behavior. Longer-term mark-recapture studies in northeastern Florida (2011–2016), have found evidence for a winter influx of transients into Mosquito Lagoon from adjacent northern estuaries [24] which also may influence abundance estimates. Seasonal variance in abundance for the southern Indian River (i.e., lower summer abundance and increased winter abundance) was similar to that observed in previous studies [21], although the cause of this variation has yet to be determined. Studies aimed at estimating IRL dolphin abundance will ultimately need to address movements of animals in and out of the study area which may be more common near the perimeters.

Although we found limited evidence for transience and temporary emigration, a prior study identified two separate dolphin communities in Mosquito Lagoon, with the northern community extending beyond the IRL boundary [60]. These findings, coupled with studies that indicate movements of IRL dolphins beyond the northern boundary [24,25], suggest this sub-basin may have higher temporary emigration and transient rates, causing greater fluctuations in abundance [21]. The majority of dolphins inhabiting the Mosquito Lagoon sub-basin have been found to exhibit strong site fidelity (71% exclusive to ML) [104], which was also observed in this study (87% of Mosquito Lagoon individuals were only observed therein). These results support Mosquito Lagoon dolphins being relatively isolated from the rest of the IRL. The current study found the greatest sub-basin exchange between the northern Indian River and the Banana River. Results are similar to a prior study which found four communities within the Indian and Banana Rivers, with one occupying a portion of the NIR and BR, and three in the SIR (this study); all overlapping adjacent basins [60]. Movements observed within the IRL coincided with previously described IRL dolphin communities, further supporting the lack of connectively between ML dolphins and the other IRL sub-basins.

Dolphin group size may be influenced by environmental factors, with smaller groups predominating in shallow estuarine waters [66,105–108]. Slightly larger mean dolphin group sizes were observed in this study than was recorded in the aerial surveys [21]. The proportion of calves (including YOY) in this study (19.2% of observations) was significantly larger than the 5.42% estimated from the aerial survey [21]. However, differences were likely due to the conservative methods utilized to define calves during aerial surveys (half the size of the adult) which would be expected to under-count calves. Our results were comparable to prior vessel-based IRL studies where calves constituted 24% of encounters [100], and to Sarasota Bay where calves constituted 21.5% [109].

### Management implications

A goal of future capture-recapture studies should be to improve stock delineation and community structure and to better quantify transient movements. Depending on management objectives of future studies, our parameter estimates could be used in simulations to identify opportunities to reduce sampling effort or increase precision. For example, variability of survival estimates increased during the current study, possibly influenced by the low number of primary periods (*n* = 4). Conducting future survey efforts over a longer time period, with an increased number of primary periods, could provide more precision in estimating temporal parameters and thus improve survival estimates. Incorporating long-term monitoring designs and comprehensive ecosystem monitoring approaches [110] may also help increase efficiencies. Lastly, the northern portion of the study area did not incorporate the full extent of the home range for dolphins in Mosquito Lagoon [21,24,25]. This could potentially cause availability bias due to temporary emigration; therefore, future studies should give further consideration to stock delineation in this area.

Effective management of the IRL dolphin stock requires data on distribution and abundance. Estimates derived herein demonstrate the feasibility of utilizing a robust design methodology and provide the first estimates of temporary emigration, transient rate, and survival for this population. The most reliable estimate of marked resident survival for IRL dolphins in this study was similar to that recorded in other bays and estuaries [31,111]. However, IRL dolphins have experienced recurring mortality peaks (2001, 2008, 2013, 2013–2015) where annual mortality peaked at 77 dolphins [37,59]. Variability in annual mortality is quite high for this population [37,59], and diminished ecosystem health [38], fisheries-related takes, and other anthropogenic activities continue to threaten this compromised dolphin population. Thus, our parameter estimates will provide critical guidance to stock management.

## Supporting information

S1 Fig Parameter estimates from robust design analysis of 1000 simulated data sets. Dolphin populations were set with initial size 1000, a detection parameter of 0.3, survival at 0.95, and the rate of gamma prime (ϒ’ = probability of a dolphin being unavailable for observation if unavailable in the prior primary period) and gamma double prime (ϒ” = probability of a dolphin being unavailable if available in the prior primary period) were both set at 0.1. *Mean parameter estimate = dot, median = triangle, vertical line = true parameter value.
(TIF)

S2 Fig Model averaged estimates (95%CI) of detection of marked bottlenose dolphins during Indian River Lagoon surveys (Florida east-central coast) by secondary session. Detection was calculated using Robust Design models for capture-recapture in the Indian River Lagoon Estuarine System.
(TIF)

S3 Fig Estimated abundance (total and marked) derived for bottlenose dolphins from the Indian River Lagoon, Florida (2016–2017). Marked abundance estimates include marked animals only. Total abundance estimates were adjusted for the ratio of marked: Unmarked individuals.
(TIF)

S4 Fig Estimated dolphin abundance estimated by sub-basin and primary period using closed population capture-recapture abundance models. Total dolphin abundance estimates were obtained by adjusting for the ratio of marked: Unmarked individuals observed in each sub-basin of the Indian River Lagoon (2016–2017).
(TIF)

S1 Table Results of robust design model selection. Parameters include: Apparent survival (φ), capture probability (p), the probability being unavailable for capture if the individual was available in the prior period (ϒ’’) or unavailable in the prior period (ϒ’) and were constrained constant (.) or allowed to vary by primary period (season) and/or secondary session. Models allowed apparent transient survival to be constant (φ (transient)), or to vary by primary period (φ(transient*season)). Three models (italics) had numerical estimation problems and were not included in inferential procedures.
(XLSX)

S2 Table Observed and photographed bottlenose dolphin groups (sightings) in the Indian River Lagoon, compiled by sub-basin and primary period. Primary periods as follows: Summer = 10–23 Aug. 2016, Fall = 1–5 Nov. 2016, Winter = 18 Jan.-3 Feb. 3, 2017, Spring = 17 Apr.-23 May 2017.
(XLSX)

S3 Table Estimated survival and movement parameters (95% CI) for the 12 best supported models for Indian River Lagoon bottlenose dolphins. Standard errors were calculated using profile likelihood estimation. To aid estimate comparisons, models with (a) and without (b) the transient parameter (time since first capture) are grouped. Time-specific adult survival rates (S) are presented for transients and residents, and survival estimates for time constant models are listed in the first time-specific row. Parameters include apparent survival (φ), model averaged estimates of adult survival rates (S) between primary periods, and temporary emigration: Conditional probability of an animal not being available for capture if it was available during the previous primary period (ϒ’’) or not available (ϒ’).
(XLSX)

S4 Table Population closure test for Indian River Lagoon dolphins suggesting population closure for all primary periods except for spring. Test subcomponent statistics indicated lack of closure in spring was due to losses between secondary sessions one and two.
(XLSX)

S5 Table Estimated detection (p) (95% CI) of marked bottlenose dolphins by sub-basin and seasonal replicate (= secondary session) during Indian River Lagoon surveys (Florida east-central coast). Detection was estimated using closed capture-recapture models for Indian River Lagoon dolphins (2016–2017).
(XLSX)

S6 Table Sighting history for thirteen bottlenose dolphins sighted only once in Mosquito Lagoon (considered potential transients). Sighting data were collected between 2008 and the end of the current study. All animals listed had prior sightings north of the Indian River Lagoon in the Halifax River. *Indicate animals known to range north into the Jacksonville Estuarine System stock.
(XLSX)

S7 Table Marked adult dolphins sighted during secondary sessions and subsequently recovered deceased during the study. (XLSX)

S1 Text Simulation study methods and results. (DOCX)
