# Supplementary figures and images for: Abundance and demography of common bottlenose dolphins (Tursiops truncatus truncatus) in the Indian River Lagoon, Florida: A robust design capture-recapture analysis
Source: PLoS One. 2021 Apr 28;16(4):e0250657. doi: 10.1371/journal.pone.0250657 (PMC8081176; doi:10.1371/journal.pone.0250657)

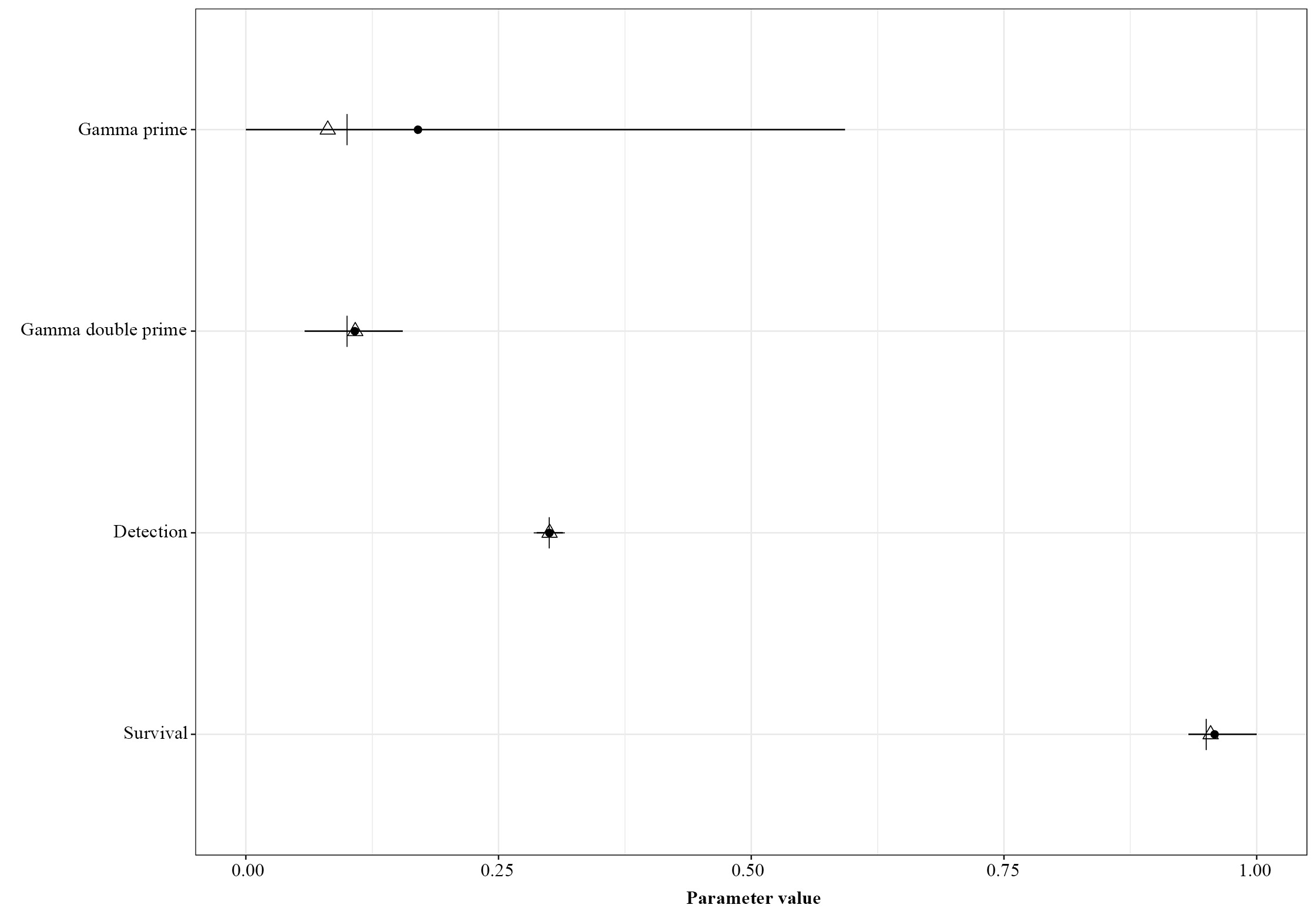

Supplement: S1 Fig — Dolphin populations were set with initial size 1000, a detection parameter of 0.3, survival at 0.95, and the rate of gamma prime (ϒ’ = probability of a dolphin being unavailable for observation if unavailable in the prior primary period) and gamma double prime (ϒ” = probability of a dolphin being unavailable if available in the prior primary period) were both set at 0.1. *Mean parameter estimate = dot, median = triangle, vertical line = true parameter value. (TIF) [file pone.0250657.s001.tif]

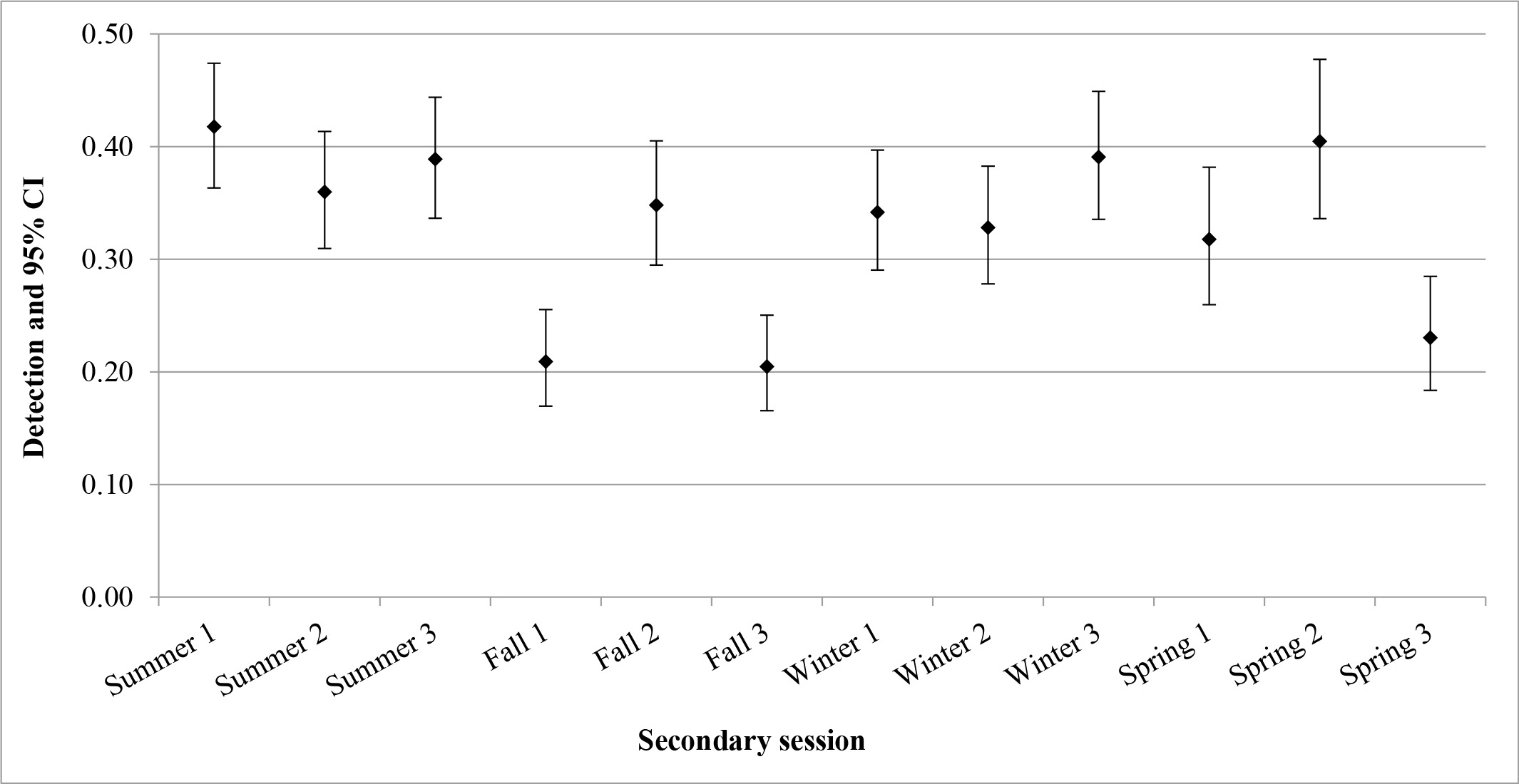

Supplement: S2 Fig — Detection was calculated using Robust Design models for capture-recapture in the Indian River Lagoon Estuarine System. (TIF) [file pone.0250657.s002.tif]

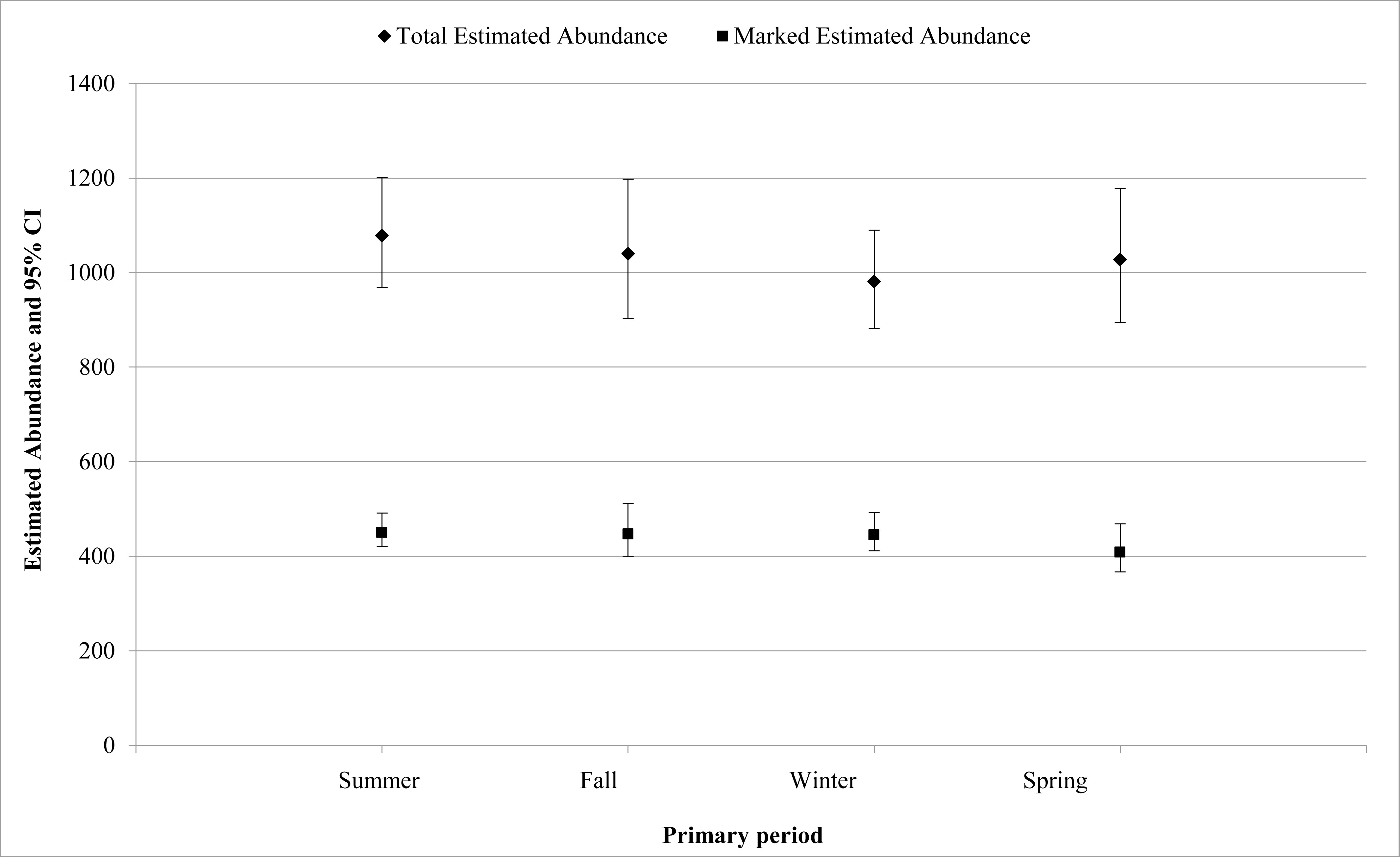

Supplement: S3 Fig — Marked abundance estimates include marked animals only. Total abundance estimates were adjusted for the ratio of marked: Unmarked individuals. (TIF) [file pone.0250657.s003.tif]

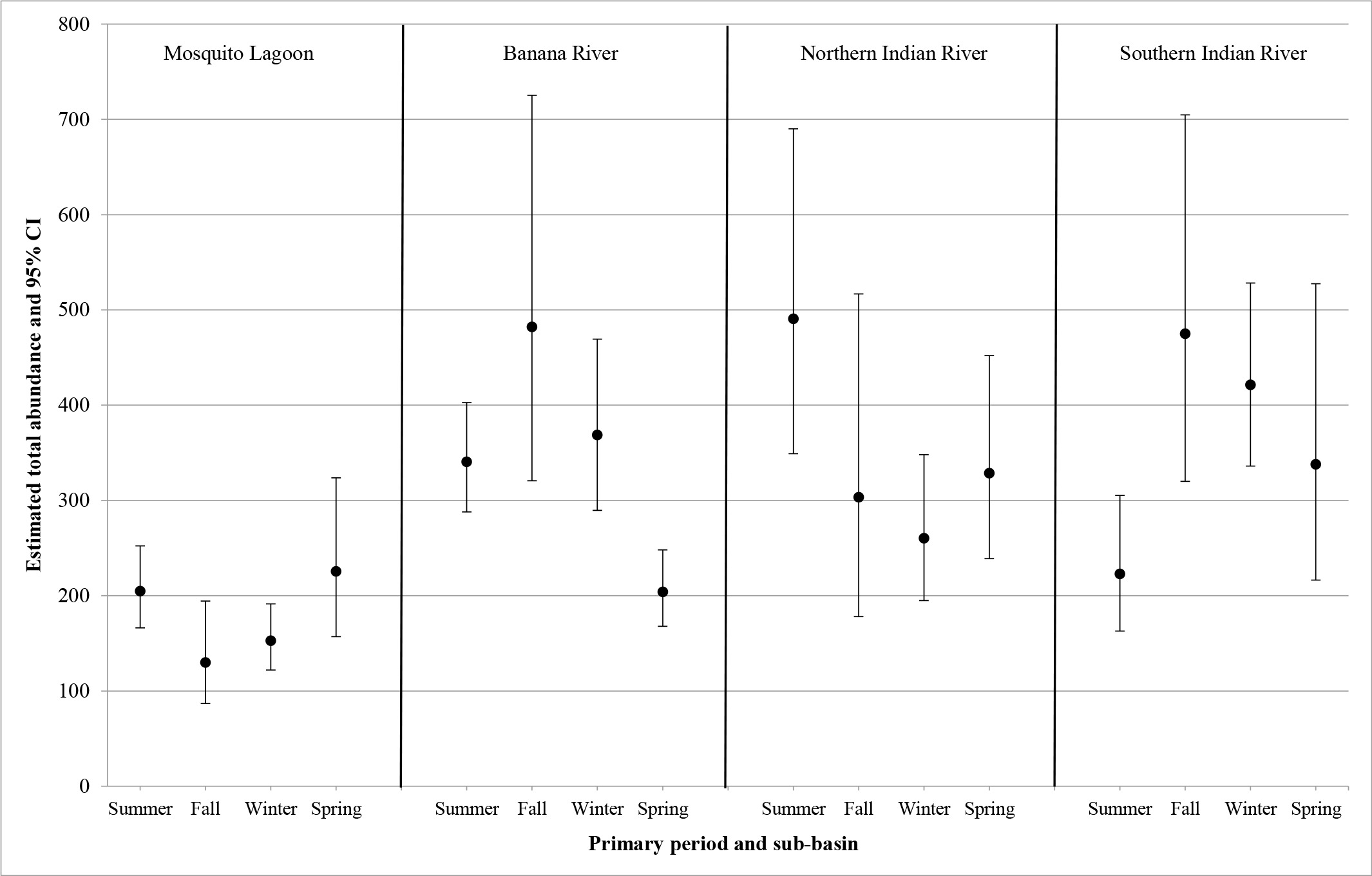

Supplement: S4 Fig — Total dolphin abundance estimates were obtained by adjusting for the ratio of marked: Unmarked individuals observed in each sub-basin of the Indian River Lagoon (2016–2017). (TIF) [file pone.0250657.s004.tif]
