# Supplementary material for: Abundance and demography of common bottlenose dolphins (Tursiops truncatus truncatus) in the Indian River Lagoon, Florida: A robust design capture-recapture analysis
Source: PLoS One. 2021 Apr 28;16(4):e0250657. doi: 10.1371/journal.pone.0250657 (PMC8081176; doi:10.1371/journal.pone.0250657)
Supplement: S1 Text — (DOCX) [file pone.0250657.s012.docx]

**S1 Text. Simulation study methods and results.**

Complex mark-recapture models with many parameters are required to account for sources of heterogeneity inherent in resighting free-ranging dolphins. For example, allowing capture probabilities to vary temporally requires additional parameters that are not directly related to abundance estimation, but are required to avoid bias and improve abundance precision. A considerable difficulty in designing mark-recapture studies is determining the amount of data required to yield precise estimates. Furthermore, it is important to understand the potential effects of heterogeneity and associated bias on abundance estimates, therefore simulation and data analysis are useful tools. In order to ensure a successful study design, a simulation study was conducted prior to the start of the study. Existing photo-ID data [19,60,104] and expert opinion were used to estimate parameter bounds for IRL dolphins (e.g., abundance by sub-basin and season, group size/structure, home range, transient rate) and for parameters associated with survey effort (e.g., capture probability and associated heterogeneity). Using R [77], 1,000 survey simulations were conducted for a population of 1000 dolphins, over four primary periods (seasons) each containing three replicate surveys (secondary sessions). The detection parameter was set at 0.3 for all samples, survival was set at 0.95 for all primary periods and both the rate of a resident leaving and remaining outside of the sample area was set at 0.1 for all primary periods. Each simulated data set was analyzed using a Robust Design mark-recapture model with survival and detection constant across time and Markovian emigration with constant rate parameters across time. Resulting abundance estimates were compared to the generating parameters to assess bias and precision, to provide a tool for planning effort levels required to achieve the desired precision, and to minimize the risk of biased estimates while allowing for the available survey effort. Seven of the 1000 simulations were discarded because of numerical estimation problems in Mark. Parameter estimates across simulations indicated that accurate estimation of detection, survival, and movement (temporary emigration) parameters could be achieved using the study design (S1 Fig.).
